# Data on mobile phone use, adaptability and adult attachment among college students in China

**DOI:** 10.1016/j.dib.2022.108397

**Published:** 2022-06-19

**Authors:** Xiaorui Liu, Tour Liu, Xinyang Liu, Xurong Lu, Yuxin Li

**Affiliations:** a Key Research Base of Humanities and Social Sciences of the Ministry of Education, Academy of Psychology and Behavior, Tianjin Normal University, Tianjin, China; b Faculty of Psychology, Tianjin Normal University, Tianjin, China; c Tianjin Social Science Laboratory of Students' Mental Development and Learning Tianjin, China

**Keywords:** Mobile phone use, Nomophobia, Mobile phone addiction, Adaptability, Adult attachment, College students

## Abstract

Mobile phone use brings convenience to people's social communication and leisurely experience. While excessive mobile phone use also leads to problematic mobile phone use such as mobile phone addiction and nomophobia which has serious harm. For college students who have just entered college, the adaptability to college life and the level of adult attachment might affect mobile phone use. Therefore, it is necessary to study the relationships among mobile phone use, adaptability and adult attachment among college students in China. The data in this article could help researchers explore the mechanism between the mobile phone use, adaptability and adult attachment and had a deeper comprehension to the impact factor of mobile phone use among college students in China. Dataset provided in this article included 673 college students recruited from different grades in Tianjin Normal University. Among the participants, there were 138 males (20.5%) and 535 females. Fifty participants completed their questionnaires as a paper-pencil version in a classroom, there were 389 participants completed paper-pencil version in total and other 284 participants completed online surveys through the Wen Juan Xing App (https://www.wjx.cn). They took Nomophobia Scale for Chinese (NMP-C), Mobile Phone Addiction Tendency Scale (MPATS), Freshmen Adaptation Inventory (FAI) and Chinese of Experiences in Close Relationships Inventory (ECR-C) to measure college students’ mobile phone use, adaptability and adult attachment in China, the missing values of these items were imputed by EM method due to the missing values were missing completely at random(MCAR). All the instruments for data collection were in the Chinese version. In addition, a .csv file consists of major variables we used were included as a supplementary material on the Zenodo Repository [1]. We used SPSS to perform descriptive statistical analysis and MPLUS to carry out lasso regression analysis with the collected data. For a discussion of the findings based on the dataset please see the article: The effect of college students’ adaptability on nomophobia based on lasso regression [2].

## Specifications Table

**Table:** 

Subject	Psychology
Specific subject area	Psychology (General)
Type of data	Microsoft Excel Comma Separated value document(.csv), Table, Figure
How data were acquired	Questionnaires, include a combination of offline paper-pencil surveys and online surveys through the Wen Juan Xing App (https://www.wjx.cn)
Data format	Raw, Analyzed
Parameters for data collection	Background: College students who used their mobile phones for a long time every day in ChinaVariables: Nomophobia, Mobile phone addiction, Learning adaptation, Professional adaptation, Homesickness adaptation, Interpersonal adaptation, Emotional adaptation, Economic adaptation, Attachment avoidance, Attachment anxietyEnrollment and Sampling method: We distribute questionnaires online and in classrooms, and subjects fill out voluntarily
Description of data collection	This data collection was carried out in 2019 before the outbreak of COVID-19, using a combination of offline surveys and onine surveys. Fifty participants completed their questionnaires as a paper-pencil version in a classroom, there were 389 participants completed paper-pencil version in total and other 284 participants completed online surveys through the Wen Juan Xing App (https://www.wjx.cn). In the online survey, the IP address of the device was recorded to prevent multiple people from participating. All participants were informed of the study purpose and provided consent to participate.
Data source location	Tianjin Normal University, Tianjin, China
Data accessibility	Respository name: zenodoData identification number: doi:10.5281/zenodo.6560781Direct link to the dataset:https://zenodo.org/record/6560781#.Yqbnoy21F0s
Related research article	J. Luo, S. Ren, Y. Li, T. Liu, The effect of college students’ adaptability on nomophobia based on lasso regression, Front. Psychiatry. 12 (2021) 641417. https://doi.org/10.3389/fpsyt.2021.641417.

## Value of the Data

• The dataset provided some important information about mobile phone use, adaptability and adult attachment among college students in China.
• These data could help researchers to explore and understand the relationships among college students’ mobile phone use, adaptability and adult attachment in China.
• These data could be used in the structural equation model (SEM), item response models, machine learning models and other analysis.
• These data were collected in the background of Chinese culture, the cross-culture and cross-sample studies would be conducted since many articles on nomophobia had been published.

## 1 Data description

The .csv file we supplied presents the data of people's situation of mobile phone use (nomophobia and mobile phone addiction), learning adaptation, professional adaptation, homesickness adaptation, interpersonal adaptation, emotional adaptation, economic adaptation, attachment avoidance and attachment anxiety among college students in China. The data was collected from online and paper-pencil questionnaires in 2019 before the outbreak of COVID-19. Five participants who had a large number of missing responses were deleted, and the remaining missing values were imputed with EM method. Finally, the first 284 rows of the .csv file are the data collected online, the last 389 rows of the .csv file are the data collected by paper-pencil. We provided the Chinese-version questionnaires and its translated version as supplementary files. For a further discussion of the major finding based on the dataset please see the article: The effect of college students’ adaptability on nomophobia based on lasso regression [2].

**Fig. 1:**
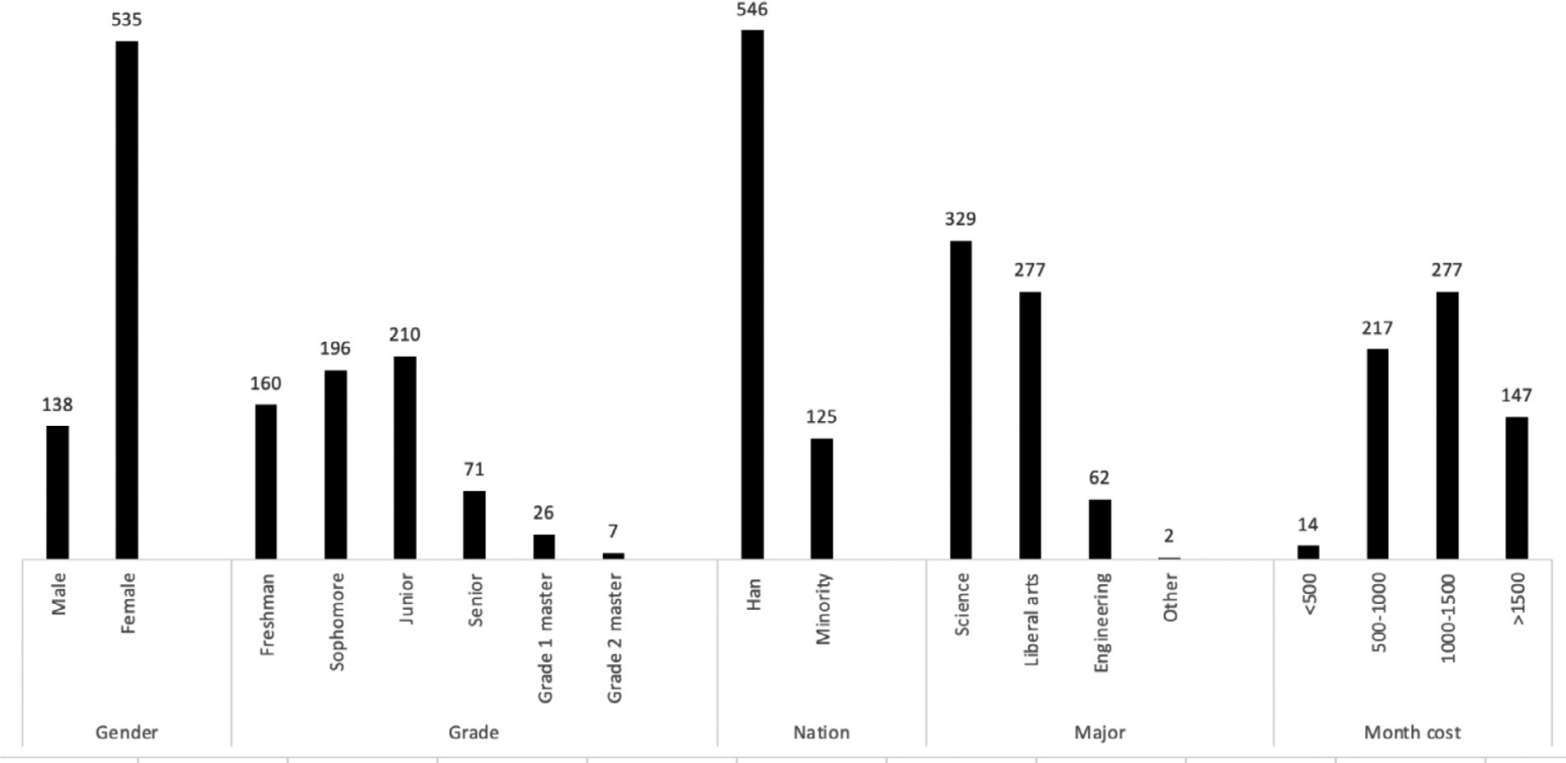
Descriptive results of the demographic variables
- the Table 1 showed the descriptive statistics for nomophobia and mobile phone addiction.

- the Fig. 1 showed the descriptive results of the demographic variables in this dataset.

**Table 1:** Descriptive statistic results of nomophobia and mobile phone addiction.

Variables	N	Minimum	Maximum	Mean	SD
1 Nomophobia Scale for Chinese(NMP-C)	673	16.00	112.00	65.42	19.30
2 Fear of being unable to obtain information	673	4.00	28.00	16.65	5.09
3 Fear of losing convenience	673	4.00	28.00	16.86	5.84
4 Fear of losing contact	673	4.00	28.00	16.89	5.96
5 Fear of losing the Internet connection	673	4.00	28.00	15.04	6.01
6 Mobile Phone Addiction Tendency Scale(MPATS)	673	16.00	80.00	43.92	11.14
7 Withdrawal symptoms	673	6.00	30.00	17.42	4.72
8 Salience	673	4.00	20.00	10.25	3.16
9 Social comfort	673	3.00	15.00	8.56	2.81
10 Mood change	673	3.00	15.00	7.68	2.59
- the Table 2 showed the descriptive statistics for adaptability and adult attachment.

**Table 2:** Descriptive statistic results of adaptability and adult attachment.

Variables	N	Minimum	Maximum	Mean	SD
1 Freshmen Adaptation Inventory(FAI)	673	44.00	139.00	94.34	13.80
2 Learning adaptation	673	4.00	24.00	15.13	3.69
3 Professional adaptation	673	4.00	24.00	16.05	4.39
4 Homesickness adaptation	673	4.00	24.00	12.93	4.47
5 Interpersonal adaptation	673	4.00	24.00	17.65	3.58
6 Emotional adaptation	673	4.00	24.00	15.95	4.02
7 Economic adaptation	673	4.00	24.00	16.63	4.08
8 Chinese of Experiences in Close Relationships Inventory(ECR-C)	673	56.00	189.00	128.03	24.38
9 Attachment avoidance	673	21.00	108.00	61.36	14.96
10 Attachment anxiety	673	18.00	108.00	66.67	17.35
- the Table 3 showed the correlations among four scales.

**Table 3:** Correlation matrix among four scales.

	1	2	3	4
1 Nomophobia Scale for Chinese(NMP-C)	1	.637⁎⁎	-.283⁎⁎	.325⁎⁎
2 Mobile Phone Addiction Tendency Scale(MPATS)		1	-.496⁎⁎	.465⁎⁎
3 Freshmen Adaptation Inventory(FAI)			1	-.484⁎⁎
4 Chinese of Experiences in Close Relationships Inventory(ECR-C)				1

*Note*:

⁎⁎ *p* < .01
- the Table 4 showed the correlations among all dimensions from each scale.

**Table 4:** Correlation matrix all dimensions from each scale.

	1	2	3	4	5	6	7	8	9	10	11	12	13	14	15	16
1 Fear of being unable to obtain information	1	.665⁎⁎	.509⁎⁎	.591⁎⁎	.501⁎⁎	.360⁎⁎	.266⁎⁎	.425⁎⁎	-.146*	-.131⁎⁎	-.123⁎⁎	-.092*	-.267⁎⁎	-.095*	.087*	.286⁎⁎
2 Fear of losing convenience		1	.617⁎⁎	.627⁎⁎	.560⁎⁎	.401⁎⁎	.331⁎⁎	.489⁎⁎	-.137⁎⁎	-.073	-.152⁎⁎	-.059	-.253⁎⁎	-.113⁎⁎	.085*	.338⁎⁎
3 Fear of losing contact			1	.662⁎⁎	.559⁎⁎	.336⁎⁎	.225⁎⁎	.402⁎⁎	-.045	-.031	-.273⁎⁎	-.001	-.172⁎⁎	-.075	-.011	.280⁎⁎
4 Fear of losing the Internet connection				1	.643⁎⁎	.500⁎⁎	.340⁎⁎	.538⁎⁎	-.140⁎⁎	-.130⁎⁎	-.167⁎⁎	-.109⁎⁎	-.254⁎⁎	-.190⁎⁎	.117⁎⁎	.393⁎⁎
5 Withdrawal symptoms					1	.684⁎⁎	.541⁎⁎	.676⁎⁎	-.174*	-.179⁎⁎	-.248⁎⁎	-.136⁎⁎	-.349⁎⁎	-.208⁎⁎	.084*	.438⁎⁎
6 Salience						1	.475⁎⁎	.645⁎⁎	-.340⁎⁎	-.251⁎⁎	-.229⁎⁎	-.191⁎⁎	-.369⁎⁎	-.269⁎⁎	.181⁎⁎	.380⁎⁎
7 Social comfort							1	.467⁎⁎	-.177⁎⁎	-.141⁎⁎	-.149⁎⁎	-.187⁎⁎	-.425⁎⁎	-.254⁎⁎	.204⁎⁎	.376⁎⁎
8 Mood change								1	-.203⁎⁎	-.179⁎⁎	-.218⁎⁎	-.204⁎⁎	-.351⁎⁎	-.300⁎⁎	.201⁎⁎	.452⁎⁎
9 Learning adaptation									1	.478⁎⁎	-.106⁎⁎	.213⁎⁎	245⁎⁎	.100⁎⁎	-.161⁎⁎	-.121⁎⁎
10 Professional adaptation										1	-.083*	.286⁎⁎	226⁎⁎	.114⁎⁎	-.102⁎⁎	-.105⁎⁎
11 Homesickness adaptation											1	-.030	.234⁎⁎	.250⁎⁎	-.084*	-.274⁎⁎
12 Interpersonal adaptation												1	.353⁎⁎	.157⁎⁎	-.191⁎⁎	-.147⁎⁎
13 Emotional adaptation													1	.455⁎⁎	-.283⁎⁎	-.462⁎⁎
14 Economic adaptation														1	-.266⁎⁎	-.282⁎⁎
15 Attachment avoidance															1	.134⁎⁎
16 Attachment anxiety																1

*Note*:

⁎ *p* < .05,

⁎⁎ *p* < .01

## 2 Experimental design, materials, and methods

### 2.1 Participants

The data presented in this article were collected from 673 college students (five cases were deleted for excessive missing values) in China. Among them, there were 138 males and 535 females. The distribution of the survey results of males and females did not change statistically. Demographic information such as grade, gender, nation, major, cost was presented in Fig. 1 Table 1.

### 2.2 Questionnaires

#### 2.2.1 Nomophobia scale for Chinese (NMP-C)

Nomophobia was measured by the 16-item Nomophobia Scale (Chinese version). Ren, Guli, and Liu revised the original Nomophobia Questionnaire by using structure equation model (ESEM) and polytomous item response model to fit NMP-C [3]. The scale involved four factors: fear of being unable to obtain information (4 items), fear of losing convenience (4 items), fear of losing contact (4 items) and fear of losing the Internet connection (4 items). This scale could measure college students’ nomophobia. Items were measured on a 7-point Likert scale (ranging from 1 = Not meet at all to 7 = Completely in conformity with). Higher score indicated higher level of the nomophobia. In the present study, the internal consistency coefficient (α) of four dimension ranged from 0.867 to 0.916 and the internal consistency coefficient (α) of the whole scale was 0.948 which has good reliability and validity. Cronbach's α for the whole scale was 0.931 and for the four dimensions were ranged from 0.789 to 0.901, the ω of the whole scale was 0.931 in this study.

#### 2.2.2 Mobile phone addiction tendency scale (MPATS)

Mobile phone addiction was measured by the 16-item Mobile Phone Addiction Tendency Scale (Chinese version). This scale was developed by Xiong, Zhou, Chen, You and Zhai [4]. The scale was composed of four factors, including withdrawal symptoms (6 items), salience (4 items), social comfort (3 items) and mood change (3 items). This scale could measure college students’ mobile phone addiction. Items were measured on a 5-point Likert scale (ranging from 1 = Very inconsistent to 5 = Very well suited to). Higher score indicated higher level of the mobile phone addiction. Previous studies found this scale reliable and valid. The internal consistency coefficient (α) of the whole scale was 0.830 and the four dimensions ranged from 0.810 to 0.920. The result of Confirmatory Factor Analysis showed that the model had good fitting indices (CFI=0.960, RMSEA=0.070, NFI=0.940, IFI=0.960, RFI=0.930). In addition, researchers have used MPATS in other studies, also proved its high construct validity (CFI=0.920, TLI=0.940, RMSEA=0.070, IFI=0.960) [5].

#### 2.2.3 Freshmen adaptation inventory (FAI)

Adaptability was measured by the 24-item Freshmen Adaptation Inventory (Chinese version). This scale was originally developed for freshmen by Cao et al. [6], and it was revised by Luo et al. [7]. The scale involved six factors: learning adaptation (4 items), professional adaptation (4 items), homesickness adaptation (4 items), interpersonal adaptation (4 items), emotional adaptation (4 items) and economic adaptation (4 items). Items from the scale were rated on a 6-point Likert scale (ranging from 1 = Very inconsistent to 6 = Very well suited to). This scale could measure college students’ adaptability and the higher scores indicates the better adaptability. Cronbach's α for the whole scale was 0.843 and for the four dimensions were ranged from 0.738 to 0.905 in this study, the ω of the whole scale was 0.795.

#### 2.2.4 Chinese of experiences in close relationships inventory (ECR-C)

Adult attachment was measured by Experiences in Close Relationships Inventory (Chinese version). This scale was originally developed for freshmen by Brennan et al. [8], and it was revised by Li et al. [9]. There were 36 items, containing two dimensions: attachment avoidance and attachment anxiety, each with 18 items. The reverse scoring items were scored reversely (item3,15,19,22,25,27,29,31,33,35). Then calculated the mean of attachment avoidance and attachment anxiety respectively. Finally calculated the scores of the four attachment styles according to Fisher's linear discriminant functions if necessary. This scale could reflect people's level of adult attachment. Items were measured on a 7-point Likert scale (ranging from 1 = strongly disagree to 7 = strongly agree). The scale showed good reliability and validity. The internal consistency coefficient (α) of the two factors was 0.82 and 0.77 respectively and the test-rest coefficients were 0.71 and 0.72 respectively.

### 2.3 Statistical analysis

The results of descriptive statistics (*Mean* and *SD*) and correlations among the total scores of major variables in the questionnaires are presented in Tables 1–4.

## Ethics Statement

This study was approved by the ethics committee of Tianjin University (XL2020-12). The questionnaire was anonymous. Age was recorded but not provided, because together with other information (e.g., gender, grade, and nation) it could expose personal identity. All participants (no minors included) were informed of the study purpose and provided consent.

## CRediT Author Statement

**Liu Xiaorui:** Data curation, Writing – original draft, Investigation, Writing – review & editing; **Liu Tour:** Supervision, Project administration; **Liu Xinyang:** Resources, Writing – review & editing; **Lu Xurong:** Resources, Writing – review & editing; **Li Yuxin:** Resources, Writing – review & editing.

## Declaration of Competing Interest

The authors declare that they have no known competing financial interests or personal relationships that could have appeared to influence the work reported in this paper.

## Appendix A. Supplementary material

Supplementary materials to this article can be found online at https://zenodo.org/record/6560781#.Yqbnoy21F0s

## Appendix B Supplementary materials

Image, application 1

Image, application 2
