# Supplementary material for: Data on mobile phone use, adaptability and adult attachment among college students in China
Source: Data Brief. 2022 Jun 19;43:108397. doi: 10.1016/j.dib.2022.108397 (PMC9240984; doi:10.1016/j.dib.2022.108397)
Supplement: Supplementary file 1 [file mmc1.docx]

**中文版施测用的问卷**

**NMP（无手机恐惧量表）**

1.如果不能通过我的手机持续访问信息，我会感到不舒服。

2.当我想要在我手机上查看信息却无法查看时，我会很生气。

3.不能在我的手机上获得新闻（例如，发生的事情，天气等）会让我感到紧张。

4.当我想要使用我手机和/或它的功能却不能使用时，我就会很生气。

5.手机电量用完会让我感到害怕。

6.如果手机达到了每月的数据流量限制，我会感到恐慌。

7.如果手机没有数据信号或者无法连接到无线网络，我会不停地检查。

8.如果我不能使用我的手机，我会害怕陷入困境。

9.当手机不在身边导致家人和/或朋友无法联系到我时，我会很担心。

10.当手机不在身边导致不能接收短信和电话时，我会感到紧张。

11.当手机不在身边导致无法与家人和/或朋友保持联系时，我会很着急。

12.当手机不在身边导致我不知道是否有人想要联系我时，我会感到很紧张。

13.当手机不在身边导致我与网络脱离时，我会很紧张。

14.当手机不在身边导致我无法赶得上社交媒体和在线网络的发展时，我会感到不舒服。

15.当手机不在身边导致我不能检查网络连接和在线网络更新的通知时，我就会觉得难受。

16.当手机不在身边导致我无法检查我的 QQ、微信、电子邮件等的信息时，我会感到焦虑。

**MPATS（手机成瘾倾向量表）**

1.一段时间没有带手机我会马上去查阅是否有短信/未接来电。

2.我宁愿选择手机聊天，不愿直接面对面交流。

3.在等人的时候我总是频发打电话问对方身在何处，如果不打就焦急难耐。

4.如果很长时间没用手机，我会觉得难受。

5.课堂上我会因为电话和短信而不能专心听讲。

6.如果没有手机我会感到孤独。

7.用手机与他人交流时，我感到更自信。

8.一段时间手机铃声不响，我会感到不适应，并下意识看一眼手机是否有未接电话/短信。

9.我经常有“我的手机铃声响了/我的手机在震动”的幻觉。

10.电话多，短信多我会觉得生活更充实。

11.我经常害怕手机自动关机。

12.手机是我的一部分，一旦减少，就觉得失去了什么似的。

13.同学朋友常说我太过于依赖手机。

14.当手机经常连不上线，收不到信号时我会焦虑并且脾气变得暴躁起来。

15.课堂上，我会经常主动把注意力集中在手机上而影响听课。

16.我觉得用手机跟他人交流更舒适。

**ECR(亲密关系经历量表-成人依恋)**

1.总的来说，我不喜欢让恋人知道自己内心深处的感觉。

2.我担心会被抛弃。

3.我觉得和恋人亲近是一件惬意的事情。

4.我很担心我的恋爱关系。

5.当恋人开始亲近我时，我感到不由自主地想逃避。

6.我担心恋人不会像我在乎他/她那样在乎我。

7.当恋人很想亲近我时，我会感到不舒服。

8.我非常担心会失去恋人。

9.当我对恋人敞开心扉时，我感到不舒服。

10.我常常希望恋人对我的感情和我对他/她的感情一样强烈。

11.我想亲近我的恋人，但我又总是退缩。

12.我经常有想要与恋人融为一体的感觉，但这常常把他/她吓跑。

13.当恋人与我太亲近时，我会感到紧张。

14.我害怕一个人独处。

15.当和恋人分享我的个人感觉和想法时，我感到很舒服。

16.我想跟恋人非常亲密的愿望，有时会把恋人吓跑。

17.我尽量避免与恋人太过亲近。

18.我需要我的恋人一再的保证他/她是爱我的，这样我才感到安心。

19.我感到与恋人亲近是比较容易的。

20.我感到我有时会强迫恋人对我表示出更多的感情和责任。

21.我发现要让我依赖恋人是件很困难的事。

22.我并不担心会被恋人抛弃。

23.我并不喜欢和恋人太过于亲近。

24.如果无法得到恋人的注意和关心，我会心烦意乱或者生气。

25.我与恋人无所不谈。

26.我感到恋人并不愿意像我所想的那样跟我亲近。

27.我经常与恋人讨论我所遇到的问题以及我关心的事情。

28.如果我还没有恋人的话，我就会感到有点焦虑和不安全。

29.我感觉依赖恋人很舒服。

30.当恋人不像我所希望的那样在我身边时，我感到很受挫。

31.我不在意从恋人那里寻求安慰、听取建议和得到帮助。

32.如果我需要恋人，但他/她不在我身边时，我会感到很沮丧。

33.在我有需要的时候向恋人求助是有用的。

34.当恋人不认同我时，我会觉得确实是我不好。

35.我会因为许多事向恋人寻求帮助，包括寻求安慰和得到承诺。

36.当恋人不花时间和我在一起时，我会感到怨恨。

**FAI(新生适应性问卷)**

1.我能有效管理时间。

2.我喜欢我的专业。

3.进大学后我很想家。

4.我与室友相处很融洽。

5.我常常感到很孤独、寂寞。

6.我除了伙食费外就没有余钱了。

7.我可以集中精力听课。

8.我对专业学习有兴趣。

9.我好想吃家里的饭菜。

10.我和同学相处得很愉快。

11.我的心情很浮躁。

12.大学的高消费令我措手不及。

13.我能保证每天自修的时间。

14.我的专业有助于我找到理想的职业。

15.我好想回家。

16.我喜欢大学宿舍的集体生活。

17.在大学里我有些自卑。

18.由于经济原因入学后我极少添置新衣物。

19.我的学习目标很明确。

20.我喜欢我所学专业的发展前景。

21.我常翻看家人的照片。

22.我觉得大学同学关系不难相处。

23.进入大学后我有一种莫名的恐惧感。

24.我担心今后的生活费没有着落。
