# Supplementary material for: Data on mobile phone use, adaptability and adult attachment among college students in China
Source: Data Brief. 2022 Jun 19;43:108397. doi: 10.1016/j.dib.2022.108397 (PMC9240984; doi:10.1016/j.dib.2022.108397)
Supplement: Supplementary file 2 [file mmc2.docx]

**Questionnaire for Data on mobile phone use, adaptability and adult attachment among college students in China**

**Basic Information**

Gender:**1** Male, **2** Female

Grade: **1** Freshmen, **2** Sophomore, **3** Junior, **4** Senior, **5** Grade 1 master, **6** Grade 2 master

Nation: **1** Han, **2** Uighur, **3** Kazakh, **4** Hui, **5** Other minority

Major: **1** Science, **2** Liberal arts, **3** Engineering **4** Other

Month cost（CNY）: **1** <500, **2** 500~1000, **3** 1000~1500 **4** >1500

**Mobile Phone Use**

# Nomophobia Scale

7-point Likert-type (from 1 = Not meet at all to 7 = Completely in conformity with)

1. I would feel uncomfortable without constant access to information through my smartphone.

2. I would be annoyed if I could not look information up on my smartphone when I wanted to do so.

3. Being unable to get the news (e.g., happenings, weather, etc.) on my smartphone would make me nervous.

4. I would be annoyed if I could not use my smartphone and/or its capabilities when I wanted to do so.

5. Running out of battery in my smartphone would scare me.

6. If I were to run out of credits or hit my monthly data limit, I would panic.

7. If I did not have a data signal or could not connect to Wi-Fi, then I would constantly check to see if I had a signal or could find a Wi-Fi network.

8. If I could not use my smartphone, I would be afraid of getting stranded somewhere.

9. If I did not have my smartphone with me, I would be worried because my family and/or friends could not reach me.

10. If I did not have my smartphone with me, I would feel nervous because I would not be able to receive text messages and calls.

11. If I did not have my smartphone with me, I would be anxious because I could not keep in touch with my family and/or friends.

12. If I did not have my smartphone with me, I would be nervous because I could not know if someone had tried to get a hold of me.

13. If I did not have my smartphone with me, I would be nervous because I would be disconnected from my online identity

14. If I did not have my smartphone with me, I would be uncomfortable because I could not stay up-to-date with social media and online networks.

15. If I did not have my smartphone with me, I would feel awkward because I could not check my notifications for updates from my connections and online networks.

16. If I did not have my smartphone with me, I would feel anxious because I could not check my email messages.

- Fear of being unable to obtain information: 1, 2, 3, 4
- Fear of losing convenience: 5, 6, 7, 8
- Fear of losing contact: 9, 10, 11, 12
- Fear of losing the Internet connection: 13, 14, 15, 16

# Mobile Phone Addiction Tendency Scale

5-point Likert-type (from 1 = Very inconsistent to 5 = Very well suited to)

1. If I haven't brought my mobile phone for a period of time, I will immediately check if there are any text messages/missed calls.

2. I would rather choose to chat on my mobile phone than face-to-face communication.

3. When I'm waiting for someone, I often call them to ask where they are and get anxious if I don't call them.

4. If I don’t use my phone for a long time, I feel uncomfortable.

5. I can't pay attention in class because of calls and text messages.

6. I would feel lonely without my mobile phone.

7. I feel more confident when communicating with others on my mobile phone.

8. When my phone doesn't ring for a while, I feel uncomfortable and subconsciously check my phone for missed calls/messages.

9. I often have hallucinations of "my cell phone is ringing/my cell phone is vibrating".

10. I feel more fulfilled when I have more phone calls and text messages.

11. I am often afraid of my mobile phone turning off automatically.

12. My mobile phone is a part of me, and when it goes away, I feel like I've lost something.

13. My classmates and friends often say that I rely too much on my phone.

14. When the mobile phone is often disconnected and cannot receive the signal, I get anxious and become irritable.

15. In class, I often take the initiative to focus on the mobile phone and affect the class.

16. I find it more comfortable to communicate with others on my mobile phone.

- Withdrawal symptoms: 1, 4, 6, 8, 10, 12
- Salience: 5, 9, 13, 15
- Social comfort: 2, 7, 16
- Mood change: 3, 11, 14

**Adaptability**

# Freshmen Adaptation Inventory

6-point Likert-type (1 = Not meet at all to 6 = Completely in conformity with)

1. I can manage time effectively.

2. I like my major.

3.* I missed home a lot after entering university.

4. I get along well with my roommate.

5.* I often feel very lonely.

6. *I don't have any money left except for boarding fee.

7. I can concentrate on listening to the class.

8. I am interested in professional learning.

9.* I'm dying for home-cooked food.

10. I get on well with my classmates.

11.* My mood is very impetuous.

12. *I was caught off guard by the high consumption in university.

13. I can guarantee the time for self-study every day.

14. My major helps me find my ideal career.

15.* I really want to go home.

16. I like the group life in university dormitory.

17.* I have a low self-esteem in college.

18. *Due to financial reasons, I seldom bought new clothes after entering school.

19. My learning goals are clear.

20. I like the development prospects of my major.

21.* I often look through the photos of my family.

22. I think it’s not difficult to get along with college classmates.

23. *I had an inexplicable sense of fear after entering college.

24. *I am worried that my living expenses will not be paid in the future.

*: Reverse scoring

- Learning adaptation: 1, 7, 13, 19
- Professional adaptation: 2, 8, 14, 20
- Homesickness adaptation: 3, 9, 15, 21
- Interpersonal adaptation: 4, 10, 16, 22
- Emotional adaptation: 5, 11, 17, 23
- Economic adaptation: 6, 12, 18, 24

**Adult Attachment**

# Experiences in Close Relationships Inventory

7-point Likert-type (from 1 = strongly disagree to 7 = strongly agree)

1. I prefer not to show aromantic partner how I feel deep down.

2. I worry about being abandoned.

3.* I am very comfortable being close to romantic partners.

4. I worry a lot about my relationships.

5. Just when my romantic partner starts to get close to me I find myself pulling away.

6. I worry that romantic partners won’t care about me as much as I care about them.

7. I get uncomfortable when my romantic partner wants to be very close.

8. I worry a fair amount about losing my romantic partner.

9. I don't feel comfortable opening up to romantic partners.

10. I often wish that my romantic partner's feelings for me were as strong as my feelings for him/her.

11. I want to get close to my romantic partner, but I keep pulling back.

12. I often want to merge completely with romantic partners, and this sometimes scares them away.

13. I am nervous when romantic partners get too close to me.

14. I worry about being alone.

15.* I feel comfortable sharing my private thoughts and feelings with my romantic partner.

16. My desire to be very close sometimes scares my romantic partner away.

17. I try to avoid getting too close to my romantic partner.

18. I need a lot of reassurance that I am loved by my romantic partner.

19. *I find it relatively easy to get close to my romantic partner.

20. Sometimes I feel that I force my romantic partners to show more feeling, more commitment.

21.I find it difficult to allow myself to depend on romantic partners.

22. *I do not often worry about being abandoned.

23. I prefer not to be too close to romantic partners.

24. If I can't get my romantic partner to show interest in me, I get upset or angry.

25. *I tell my romantic partner just about everything.

26. I find that my romantic partner doesn't want to get as close as I would like.

27.* I usually discuss my problems and concerns with my romantic partner.

28. When I'm not involved in a relationship, I feel somewhat anxious and insecure.

29. *I feel comfortable depending on romantic partners.

30. I get frustrated when my romantic partner is not around as much as I would like.

31. *I don't mind asking romantic partners for comfort, advice, or help.

32. I get frustrated if romantic partners are not available when I need them.

33. *It helps to turn to my romantic partner in times of need.

34. When romantic partners disapprove of me, I feel really bad about myself.

35. *I turn to my romantic partner for many things, including comfort and reassurance.

36. I resent it when my romantic partner doesn’t spend time with me

*: Reverse scoring

- Attachment avoidance: 1, 3, 5, 7, 9, 11, 13, 15, 17, 19, 21, 23, 25, 27, 29, 31, 33, 35
- Attachment anxiety: 2, 4, 6, 8, 10, 12, 14, 16, 18, 20, 22, 24, 26, 28, 30, 32, 34, 36
